# Maternal fatty acid status during pregnancy versus offspring inflammatory markers: a canonical correlation analysis of the MEFAB cohort

**DOI:** 10.3389/fnut.2023.1264278

**Published:** 2023-10-19

**Authors:** Sven H. Rouschop, Agnieszka Smolinska, Marij Gielen, Renate H. M. de Groot, Maurice P. Zeegers, Antoon Opperhuizen, Frederik J. van Schooten, Roger W. Godschalk

**Affiliations:** ^1^Department of Pharmacology and Toxicology, School for Nutrition and Translational Research in Metabolism (NUTRIM), Maastricht University, Maastricht, Netherlands; ^2^Department of Epidemiology, CAPHRI Care and Public Health Research Institute, Maastricht University, Maastricht, Netherlands; ^3^Department Conditions for Life Long Learning, Faculty of Educational Sciences, Open University of the Netherlands, Heerlen, Netherlands; ^4^Nederlandse Voedsel en Warenautoriteit (NVWA), Utrecht, Netherlands

**Keywords:** childhood asthma, canonical correlation analyses (CCA), fatty acid, pregnancy, inflammation

## Abstract

The development of inflammatory lung disorders in children may be related to maternal fatty acid intake during pregnancy. We therefore examined maternal fatty acid (FA) status during pregnancy and its associations with inflammatory markers and lung conditions in the child by analyzing data from the MEFAB cohort using multivariate canonical correlation analysis (CCA). In the MEFAB cohort, 39 different phospholipid FAs were measured in maternal plasma at 16, 22 and 32 weeks of pregnancy, and at day of birth. Child inflammatory markers and self-reported doctor diagnosis of inflammatory lung disorders were assessed at 7 years of age. Using CCA, we found that maternal FA levels during pregnancy were significantly associated with child inflammatory markers at 7 years of age and that Mead acid (20:3n-9) was the most important FA for this correlation. To further verify the importance of Mead acid, we examined the relation between maternal Mead acid levels at the day of birth with the development of inflammatory lung disorders in children at age 7. After stratification for the child’s sex, maternal Mead acid levels at day of birth were significantly related with self-reported doctor diagnosis of asthma and lung infections in boys, and bronchitis and total number of lung disorders in girls. Future studies should investigate whether the importance of Mead acid in the relation between maternal FA status and inflammation and lung disorders in the child is due to its role as biomarker for essential fatty acid deficiency or due to its own biological function as pro-inflammatory mediator.

## Introduction

Inflammatory lung diseases are a major burden to global health. According to the World Health Organization (WHO), respiratory infections are the fourth leading cause of both death and burden of disease in the world (1). Similarly, asthma prevalence has increased over the past few decades (2–4), making asthma the most common chronic disease in children (5). The development of inflammatory lung disorders in children has been related to a maternal fat intake during pregnancy. Additionally, animal studies have demonstrated that maternal high fat diets during gestation predispose weanling offspring to airway inflammation, airway hyperresponsiveness, and increased susceptibility to Respiratory Syncytial Virus (RSV) infection (6, 7). Likewise, both observational and intervention studies in humans have shown that the risk of having asthma and asthma-related symptoms in offspring is negatively correlated with maternal intake of n-3 polyunsaturated fatty acids (PUFAs) (8–13). Furthermore, maternal fatty acid (FA) status during pregnancy has been linked to inflammation in general and the development of the immune system in the child (14); more specifically the n-6/n-3 PUFA ratio in the maternal diet seems to be important, with n-6 FA in the maternal diet as pro-inflammatory mediators, whereas n-3 FA were reported to have anti-inflammatory effects.

When examining maternal FA status during pregnancy and its relation with offspring health, FA levels are often estimated through the use of food frequency questionnaires (FFQs) (9, 15) rather than using objective FA biomarkers, such as plasma phospholipids. Furthermore, studies usually assess maternal FAs at a single prenatal time point (16, 17) instead of measuring the FA status throughout pregnancy. Finally, the relation between maternal FA status and offspring’s health is commonly studied by analyzing single associations between an individual exposure variable and an individual outcome parameter (9, 15–17). However, the complex etiology of biological disorders may be better studied by examining a set of exposure variables and a set of outcome parameters simultaneously (18). Therefore, the aim of this study was to examine maternal FA status and the association with inflammation and lung disorders in the child, taking these shortcomings into account.

We used data of the MEFAB cohort that was established to study the associations between maternal FA status during pregnancy and pregnancy outcomes (19). In this cohort, maternal plasma phospholipid FAs were measured at 16, 22, and 32 weeks of pregnancy, and at day of birth, and various child inflammatory markers were measured at 7 years of age. These inflammation endpoints included: total white blood cell, monocyte, granulocyte and lymphocyte count, and blood concentrations of tissue plasminogen activator (tPA), plasminogen activator inhibitor-1 (PAI-1), leptin, fibrinogen and C-reactive protein (CRP). The simultaneous correlation analysis of multiple independent variables (here: FA in maternal blood) and multiple dependent variables (here: inflammatory outcomes) can be performed by canonical correlation analysis (CCA). Although CCA is used in many research areas, such as social and behavioral research (20), bioinformatics (21), and genetics (22), CCA has not yet been used before to study the relation between maternal fatty acid status and child inflammation. Finally, we investigated whether the maternal FA concentrations during pregnancy that contributed most to the CCA analysis correlated with the self-reported doctor diagnosis of bronchitis, asthma and lung infections in the child at the age of 7 years.

## Methods

**Figure 1:**
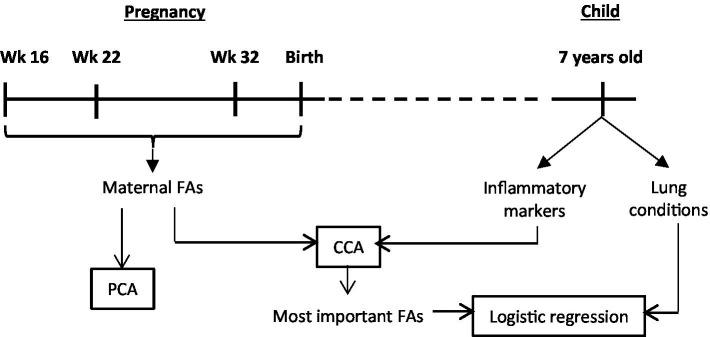
Schematic overview of the study design and analysis workflow. Wk, weeks of pregnancy; FAs, fatty acids; PCA, principal component analysis; CCA, canonical correlation analysis.

A schematic overview of the study design and analysis workflow are presented in Figure 1.

### Study design, setting and participants

Pregnant women attending one of three antenatal clinics in the south the Netherlands between 1989 and 1995 were asked to participate in the cohort. Women were eligible for participation if they were less than 16 weeks pregnant and did not suffer from any cardiovascular, neurological, renal or metabolic condition. Originally, 1,203 women were included in the MEFAB cohort. For the follow-up, 305 singletons born between 1990 and 1994 were included between 1997 and 2000. For the current analysis, only mother–child pairs were included for which there were no missing data in any of the maternal plasma fatty acid concentrations or child plasma inflammatory markers, leading to a total number of 173 mother–child pairs. The study was approved by the medical ethics committee of the University hospital Maastricht and Maastricht University. Written consent was given by pregnant women before enrolment in the initial study and again by both parents (if possible) for the follow-up study.

### Exposure variables

To measure maternal FA status during pregnancy, maternal venous blood samples were collected in EDTA tubes around 16, 22 and 32 weeks of pregnancy, and immediately after delivery. Concentrations of 39 different plasma phospholipid FAs were determined by capillary gas–liquid chromatography as described previously (23) and expressed in mg/L. See Supplementary Table S1 for a list of the FAs that were measured. Plasma was stored at –80°C until fatty acid analysis. To check whether the fatty acid profiles changed with different lengths of storage, Pearson correlation coefficients were calculated between the relative amounts of fatty acid in maternal or umbilical plasma phospholipid and storage time. Since no significant correlations were observed, an effect of storage time on the fatty acid profile was excluded. Changes in maternal fatty acid status throughout pregnancy were studied by including all 39 fatty acids in a principal component analysis (PCA) for each of the 4 time points during and directly after pregnancy.

### Outcome variables

At 7 years of age, offspring inflammatory markers were measured in plasma. These markers included: total white blood cell, monocyte, granulocyte and lymphocyte count (×10^9^/L) determined by Beckman-Coulter Gen-s (Beckman Coulter, Brea, CA, USA); tissue plasminogen activator (tPA) concentration (ng/ml) and enzyme activity (IU/ml) measured by ELISA and bioimmunoassay, respectively (Biopool International, Ventura, CA, USA); plasminogen activator inhibitor-1 (PAI-1) concentration (ng/ml); leptin concentration (μg/l) determined by RIA (Linco Research, St Charles, MO, USA); fibrinogen concentration (g/l) measured by the Clauss method (24); and C-reactive protein (CRP) concentration (mg/l) assayed with an in-house ELISA using polyclonal antibodies as catching and tagging antibodies labelled with HRP (Dako, Glostrup, Denmark). To assess the children’s history of lung disorders, their parents were asked at follow-up whether their child was ever diagnosed by a medical doctor with asthma, bronchitis, or pneumonia which was treated with antibiotics.

### Confounding variables

Information on potential confounders was obtained by using medical records and questionnaires for maternal age (years) and BMI (kg/m^2^) at study entry, duration of pregnancy (days), child sex, and child birth weight (grams). Information regarding maternal smoking and alcohol consumption during pregnancy (yes or no), breastfeeding (yes or no) with or without a combination of formula feeding, presence of pets (dog, cat, rodent or bird) at home during childhood (yes or no), and daycare attendance during first 4 years (yes or no) was obtained from questionnaires at the age of 7 years of the child. Additionally, the child’s BMI (kg/m^2^) was assessed by a nurse during a follow-up visit.

### Canonical correlation analysis

The relation between maternal FA status and child inflammatory status was studied using CCA. CCA is a method which explores the linear relationship between two multivariate data sets X and Y (Equation 1). CCA does this by making separate linear combinations (called canonical variates) of both data sets by multiplying each separate variable in X or Y with a coefficient (a so-called canonical weight) and taking the sum of these products (Equation 2). Canonical weights are chosen in such a way that the correlation between the canonical variates of X and Y is maximized. CCA develops as many pairs of canonical variates as there are variables in the smallest of the two data sets. Furthermore, all canonical variates are orthogonal (uncorrelated) of each other, to make sure that each pair of canonical variates represents a different relationship between X and Y. To test if the correlation between two canonical variates is significant, an asymptotic test using F-approximation of Wilks’ Lambda was applied. Only canonical variate pairs with a significant correlation were used for biological interpretation. Individual variables were considered to have a considerable contribution to a canonical correlation if their canonical loadings, which represent the correlation between the individual variable and the conical variate, were > |0.3|.


(1) $$X=\left(\begin{array}{c} x_{1}\\ x_{2}\\ \begin{array}{c} \ldots \\ x_{p} \end{array} \end{array}\right),Y=\left(\begin{array}{c} y_{1}\\ y_{2}\\ \begin{array}{c} \ldots \\ y_{q} \end{array} \end{array}\right)$$

(2) $$\begin{array}{c} U_{1}=a_{11}x_{1}+a_{12}x_{2}+\ldots +a_{1p}x_{p},V_{1}=b_{11}y_{1}+b_{12}y_{2}+\ldots +b_{1p}y_{p}\;\\ U_{2}=a_{21}x_{1}+a_{22}x_{2}+\ldots +a_{2p}x_{p},V_{2}=b_{21}y_{1}+b_{22}y_{2}+\ldots +b_{2p}y_{p}\;\\ \begin{array}{c} \ldots \\ U_{p}=a_{p1}x_{1}+a_{p2}x_{2}+\ldots +a_{ppx_{p}},V_{p}=b_{p1}y_{1}+b_{p2}y_{2}+\ldots +b_{pp}y_{p} \end{array} \end{array}$$

Canonical correlation analysis explores the linear relationship between two multivariate data sets X and Y (Equation 1). It does so by making separate linear combinations (canonical variates) of both data sets by multiplying each separate variable in X or Y with a canonical weight and taking the sum of these products (Equation 2).

### Model optimization

**Figure 2:**
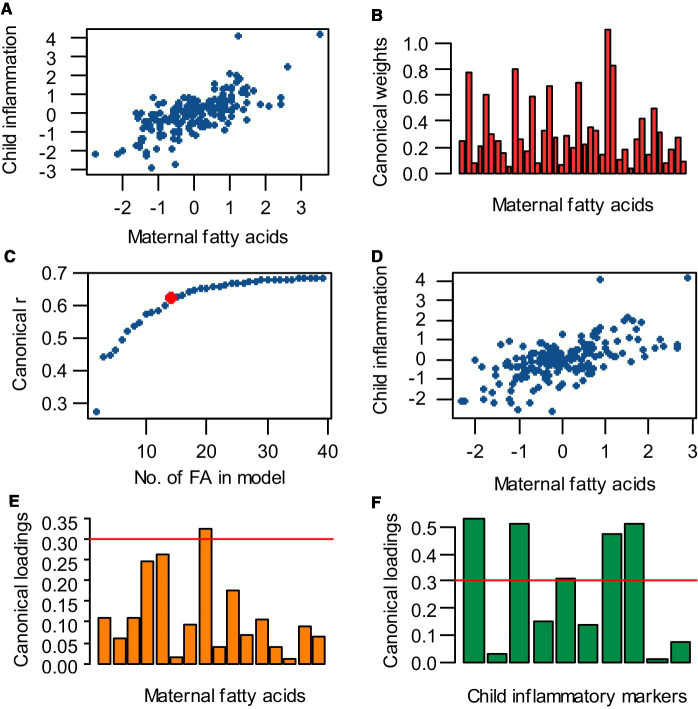
Analysis strategy for performing canonical correlation analysis (CCA), reducing the number of independent variables, obtaining an optimal model, and selecting the most important variables. **(A)** A full CCA model was obtained, containing all 39 maternal fatty acids vs. all child inflammatory markers. **(B)** To reduce the number of independent variables in the CCA model, the fatty acid variable with the lowest absolute, standardized weight was eliminated and the analysis was repeated. **(C)** The process of removing the least contributing independent variable and rerunning the analysis was iterated until an optimum was achieved between a low number of variables and a high correlation. This optimum was set at the point where the canonical correlation coefficient (r) starts decreasing with increasing intervals while cutting out independent variables (indicated by the red dot). **(D)** With the optimal number of independent variables, an optimal CCA model was made. **(E,F)** Fatty acids and inflammatory markers with an absolute loading >0.3 (cut-off indicated by the red line) were selected as most important for the canonical correlation.

First a full CCA model was obtained using all 39 maternal fatty acids as independent variables and all child inflammatory markers as dependent variables (Figure 2A). To further identify which specific fatty acids were most important for this relation, the number of independent variables in the model was reduced by deleting the fatty acid variable with the smallest contribution to the model (i.e., with the smallest absolute, standardized canonical weight) and the analysis was repeated (Figure 2B). By removing variables, information is withdrawn from the model and the correlation decreases. Therefore, this process of removing the least contributing independent variable was iterated until an optimal balance was achieved between a low number of independent variables and a high correlation (Figure 2C). This optimum was chosen to be at the point where the correlation coefficient started to visually decrease quicker or at a canonical r ≥ 0.6. With this optimal number of independent variables, an optimal CCA model was made (Figure 2D). For all optimized CCA models, the most important maternal fatty acids and child inflammatory markers were identified by selecting variables with an absolute canonical loading >|0.3| (Figures 2E,F). Potential confounders were initially not included in the analysis, but were taken into account in follow up logistic regression analyses for the occurrence of lung disorders.

### Logistic regression

The relation between maternal FA variables with a canonical loading >|0.3| and offspring lung disorders was assessed using binomial and ordinal logistic regression. The influence of potential confounders on logistic regression was determined by including the potential confounder as a covariate in the analysis. The variable was considered to be a confounder if inclusion of the confounder led to a significant covariate effect, the main effect not being significant anymore, or a change >10% of the main effect odds ratio. For all statistical analyses, *p* < 0.05 was considered significant. All statistical analyses were performed using R version 4.0.2 (R Foundation for Statistical Computing, Vienna, Austria). CCA was done with CCA package version 1.2.

## Results

### Population characteristics

Practically all mothers included in the analysis were Caucasian (99%; Table 1). On average, mothers were 30 years-old when entering the study and had a BMI of 24 kg/m^2^ and a normal pregnancy duration (279 days). Twenty percent of the mothers smoked during pregnancy, with a mean of nine cigarettes per day. Three percent consumed alcohol during pregnancy, but none specified how much. Deliveries were mostly vaginal (95%) and half of the infants were breastfed for some period of time (47%). About half of the children were female (43%). On average, children had a normal birthweight (3,362 grams) and BMI at 7 years-of-age (15 kg/m^2^).

**Table 1:** Population characteristics of the MEFAB participants included in this study (*n* = 173 mother–child pairs).

Variable	Mean (SD)	Median (IQR)
Mother		
Ethnicity (Caucasian *n*, %)	*n* = 172, 99.4%	
Age at entry study (years)	30.12 (4.14)	29.54 (5.33)
BMI at entry study (kg/m^2^)	23.56 (3.75)	23.05 (3.66)
Pregnancy		
Duration (days)	279 (11)	281 (12)
Smoking (*n*, %)	*n* = 34, 19.6%	
Cigarettes per day	9.17 (5.62)	9.50 (5.00)
Alcohol consumption (*n*, %)	*n* = 5, 2.9%	
Caesarian section (*n*, %)	*n* = 8, 4.6%	
Breastfeeding (*n*, %)	*n* = 81, 46.8%	
Child		
Sex (Female *n*, %)	*n* = 75, 43.4%	
Birthweight (grams)	3,362 (495)	3,400 (640)
BMI at 7 years*	15.47 (1.88)	15.03 (2.12)
Asthma (*n*, %)	*n* = 12, 6.9%	
Bronchitis (*n*, %)	*n* = 39, 22.5%	
Lung infection (*n*, %)	*n* = 12, 7.0%	

SD, standard deviation; IQR, interquartile range. ^*^Please note that a BMI between 13.5 and 17.6 is considered normal for children at the age of 7 years.

### Maternal plasma fatty acid status changed throughout pregnancy

**Figure 3:**
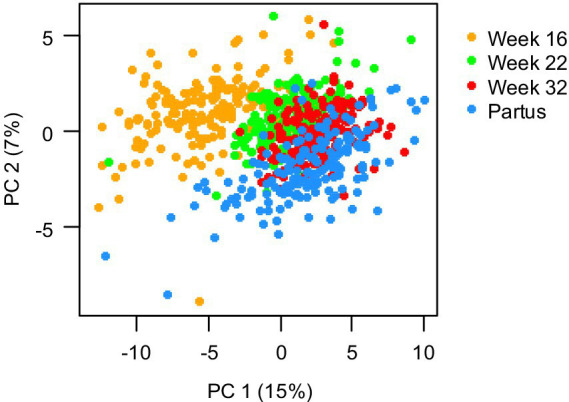
Principal component analysis of 39 maternal plasma fatty acid concentrations, measured at 16, 22, and 32 weeks of pregnancy, and at day of birth (partus). Data are presented as scatter plot of the first and second principal component (PC), covering 15 and 5%, respectively, of the total variance, with each dot representing one mother at a specific gestational time point (*n* = 173 mothers).

To assess the progression of maternal fatty acid status during pregnancy, PCA was applied to maternal plasma concentrations of 39 different FAs during pregnancy. The result from this is shown in Figure 3, with each dot representing a mother, with different colors representing different prenatal time points and the position of the dot representing the overall FA status. The gradual shift of the cloud of dots from week 16 to week 22, to week 32, to partus indicates that the overall maternal FA profile gradually changed throughout pregnancy, with the largest shift occurring between 16 and 22 weeks of pregnancy.

### Maternal fatty acid status during pregnancy correlated with child inflammatory status at 7 years of age

To assess the multivariate relation between maternal FA levels during pregnancy and inflammatory markers in the child at 7 years of age, CCA was used. Since PCA showed that the maternal fatty acid status gradually changed throughout pregnancy, a separate CCA was performed for each prenatal time point. The optimized CCA models for week 16, week 22 and week 32 of pregnancy and partus contained sixteen, fourteen, fifteen, and nineteen independent FA variables, respectively (Table 2) and all models were statistically significant (week 16: *r* = 0.60, *p* < 0.001; week 22: *r* = 0.62, *p* < 0.001; week 32: *r* = 0.64; *p* = 0.001; partus: *r* = 0.61, *p* = 0.028; Table 2). All lower-order canonical variates were not significant and thus not used for further interpretation (Supplementary Table S2). For each significant canonical variate, the most important independent and dependent variables were identified by selecting variables with an absolute canonical loading >0.3. Of these various fatty acids and inflammatory markers that were identified to be important for the different canonical correlations, Mead acid (20:3n9) and granulocyte count, respectively, occurred most often at the different time points (Table 2).

**Table 2:** Canonical correlation analysis of maternal plasma fatty acid (FA) concentrations at 16, 22, and 32 weeks of pregnancy, and at day of birth (partus) versus child plasma inflammatory markers at 7 years of age (*n* = 173 mother–child pairs).

	No. of FAs in CCA model	*r*	Value of *p*	FAs |loading| > 0.3	Inflammatory markers |loading| > 0.3
Week 16	16	0.60	<0.001	24:1n9 18:1DMA	Monocytes Granulocytes tPA activity
Week 22	14	0.62	<0.001	20:3n9	White blood cells Granulocytes PAI-1 Leptin
Week 32	15	0.64	0.001	22:2n6 16:0DMA 20:3n9	White blood cells Granulocytes tPA activity tPA concentration PAI-1
Partus	19	0.61	0.028	20:3n9 24:1n9 22:2n6 16:0DMA	White blood cells Granulocytes tPA concentration PAI-1 CRP

r, canonical correlation coefficient.

### Maternal Mead acid levels at day of birth are positively associated with the development of lung disorders in offspring

**Figure 4:**
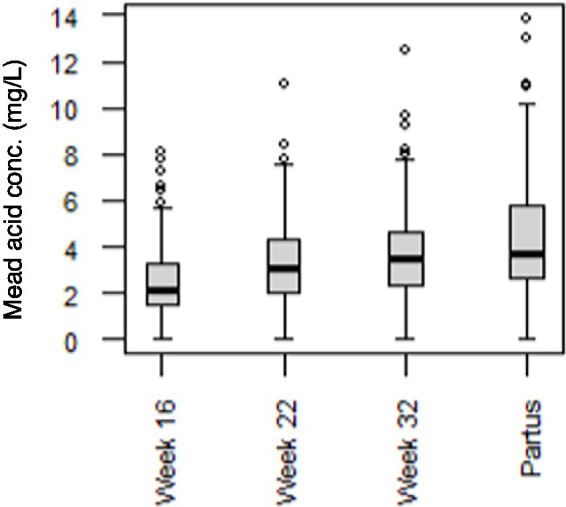
Mead acid concentrations in maternal plasma at 16, 22, and 32 weeks of pregnancy, and at day of birth (partus). Data are presented as boxplots, with dots representing values higher than 1.5 times the interquartile range above the third quartile (*n* = 173 mothers).

Since Mead acid (20:3n-9) showed a canonical loading of >|0.3| at 3 time points (week 22-partus) in the CCA, the relevance of maternal Mead acid levels during pregnancy was further assessed by examining the association between maternal plasma levels of Mead acid at day of birth and the odds of the child developing asthma, bronchitis and/or lung infection during the first 7 years of age. Only Mead acid data from the day of birth were used for this analysis, since Mead acid concentrations consistently increased throughout pregnancy, with the highest concentrations and the largest variation occurring at that specific time point (Figure 4). Using unadjusted binomial regression, a significant, positive relation was found between maternal Mead acid levels (mg/L) and self-reported doctor diagnosis of bronchitis (OR [95% CI] = 1.17 [1.01–1.34]) and lung infection (OR [95% CI] = 1.27 [1.04–1.55]), but not asthma (Table 3). Furthermore, unadjusted ordinal logistic regression showed a significant, positive association between maternal Mead acid concentrations at day of birth (mg/L) and the total number of self-reported doctor-diagnosed lung disorders that the child suffered from during the first 7 years of age (OR [95% CI] = 1.18 [1.03–1.34]; Table 3). Adjusting the logistic regression models for maternal age or BMI at study entry, gestational age, smoking or alcohol use during pregnancy, birthweight, breastfeeding, presence of pets, daycare attendance, or child BMI at 7 years of age left the effect estimate and significance of Mead acid largely unchanged (Supplementary Table S3). Stratification for child sex, however, did lead to sex-specific results. For asthma, bronchitis and number of lung conditions, associations were significant for girls (OR [95% CI] =1.77 [1.09–3.62], 1.32 [1.03–1.72], and 1.40 [1.10–1.80], respectively) but not boys, whereas for lung infection, associations were significant for boys (OR [95% CI] = 1.28 [1.02–1.63]), but not girls (Table 3).

**Table 3:** Associations between maternal plasma mead acid concentration at day of birth (mg/L) and self-reported doctor diagnosis of lung disorders in the child during the first 7 years of age (*n* = 173 mother–child pairs).

	OR	95% CI	Value of *p*
Asthma			
Unstratified	1.13	0.91–1.38	0.228
Girls	1.77	1.09–3.62	0.038
Boys	1.03	0.77–1.29	0.842
Bronchitis			
Unstratified	1.17	1.01–1.34	0.030
Girls	1.32	1.03–1.72	0.029
Boys	1.11	0.93–1.33	0.227
Lung infection			
Unstratified	1.27	1.04–1.55	0.014
Girls	1.27	0.82–1.91	0.237
Boys	1.28	1.02–1.63	0.029
Number of lung disorders			
Unstratified	1.18	1.03–1.34	0.007
Girls	1.40	1.10–1.80	0.003
Boys	1.12	0.94–1.31	0.093

Statistical analysis by binomial logistic regression (for asthma, bronchitis and lung infection) or ordinal logistic regression (for number of lung conditions). OR (odds ratio), 95% CI (confidence interval) and value of *p* are shown for both unadjusted results and results stratified for child sex.

## Discussion

The development of inflammatory lung disorders has previously been shown to be related to the total fat content or the fatty acid composition of the maternal diet during pregnancy. However, estimates of prenatal exposures and statistical analysis were often suboptimal in previous studies. Therefore, this study examined the relation between maternal FA status and child inflammation by analyzing data from the MEFAB cohort using CCA. This paper showed that maternal plasma FA levels during pregnancy were significantly associated with child inflammatory markers at 7 years of age and that Mead acid was a fatty acid that contributed most to the CCA (loading >ǀ0.3ǀ) at different time points of pregnancy (from 22 weeks until delivery). In addition, we showed that maternal Mead acid levels at day of birth were significantly correlated with self-reported asthma, bronchitis and the total number of lung conditions in girls, and with self-reported lung infection in boys.

First, we confirmed with a PCA that included 39 different FAs that the profile of maternal FAs gradually changed throughout pregnancy. This finding emphasizes the importance of assessing FA levels at consistent prenatal time points when studying the relation between maternal FA status and offspring health, rather than assessing FA exposure at varying prenatal time points, as has been the case for previous studies. For instance, Notenboom et al. measured maternal FA levels in the 34th to 36th week of gestation (16), whereas Rucci et al. measured maternal FA levels around the 20th week of gestation (17), making comparison of both studies suboptimal. Moreover, Miyake et al. assessed maternal FA consumption at study entry, irrespective of the gestational age (9), thereby potentially introducing heterogeneity in the exposure data set. The current study, in contrast, measured maternal FA levels in plasma phospholipids at 16, 22 and 32 weeks of gestation, and at day of birth, thereby covering both the second and third trimester. In addition, maternal FA were assessed in phospholipids, which represent FA intake of the past few days (25). Altogether, the exposure variables as assessed in the current study may thus be considered as a comprehensive representation of prenatal maternal FA status throughout pregnancy.

Using CCA, we examined the multivariate relation between child inflammatory markers at 7 years of age and maternal plasma FA levels at 16, 22 and 32 weeks of gestation, and at day of birth. With this approach, we found that for all prenatal time points, maternal blood concentrations of various FAs were significantly associated with child inflammatory markers at 7 years of age. Of those FA that were related to child inflammatory markers, Mead acid was identified as a significant maternal FA for these multivariate correlations at multiple time points (i.e., all time points after 22 weeks). Mead acid (20:3n-9) is an n-9 PUFA which is synthesized *in vivo* from oleic acid (18,1n-9) through elongation and desaturation by the enzymes Fatty acid desaturase 1 (Fads1), Fatty acid desaturase 2 (Fads2) and Elongation of very long chain fatty acids protein 5 (Elovl5) (26). Fads1, Fads2 and Elovl5 also elongate and desaturate the n-6 and n-3 PUFAs linoleic acid (LA, 18:2n-6) and α-linolenic acid (ALA, 18:3n-3) into their respective longer-chain derivatives, such as arachidonic acid (AA, 20:4n-6), eicosapentaenoic acid (EPA, 20:5n-3) and docosahexaenoic acid (DHA, 22:6n-3) (27). As a result of these shared metabolic pathways between n-3, n-6 and n-9 PUFAs, Mead acid synthesis is enhanced when n-3 and n-6 PUFA levels are low, making Mead acid to be considered as a biomarker for essential fatty acid deficiency (28). Next to this, Mead acid has its own biological functions. Similar to the conversion of AA into 5-hydroxyeicosatetraenoic acid (5-HETE) by 5-lipoxygenase (5-LOX) and its subsequent oxidation into 5-oxo-eicosatetraenoic acid (5-oxo-ETE) by 5-hydroxyeicosanoid dehydrogenase (5-HEDH), Mead acid is metabolized into 5-hydroxyeicosatrienoic acid (5-HETrE) and 5-oxo-eicosatrienoic acid (5-oxo-ETrE), respectively, by the same enzymes (29, 30). In accordance with these structural analogies, 5-oxo-ETrE is as potent as 5-oxo-ETE in inducing chemotaxis, surface expression of CD11b and calcium mobilization in neutrophils, as well as increasing actin polymerization in eosinophils (29). Mead acid thus may act as a pro-inflammatory mediator, thereby potentially contributing to the course of inflammatory lung disorders, such as lung infections, asthma and bronchitis. From these data, one could speculate that mothers should assure sufficient intake of essential fatty acids during pregnancy to avoid *de novo* synthesis of Mead acid.

In a canonical correlation analysis, multiple variables (here: maternal FA during and directly after pregnancy) are statistically linked to multiple dependent variables (here: inflammatory markers in the child at 7 years of age). Each independent and dependent variable has a certain contribution to the overall relationship. Next to Mead acid being identified as an important independent variable, the outcome variable that contributed most to the multivariate correlation between maternal FA status and child inflammation was granulocyte count. Granulocytes play an important role in various inflammatory lung disorders. For instance, both eosinophils and neutrophils are associated with clinical severity and pulmonary dysfunction in asthma patients (31–34). Similarly, fungal infection of the lungs is associated with pulmonary influx of eosinophils (35), whereas neutrophils contribute to early defense against pulmonary infection with bacteria (36). Mead acid may possibly affect lung conditions through its effect on granulocytes, because both eosinophils and neutrophils are involved in inflammatory lung conditions. Whether this hypothesis is correct and what molecular or cellular mechanisms would be involved in this, remains to be examined by future studies.

To further evaluate the relevance of maternal Mead acid levels during pregnancy, the association between maternal plasma levels of Mead acid at day of birth and the self-reported doctor diagnosis of lung disorders in children was examined. As a result, maternal Mead acid levels at day of birth were positively associated with the diagnosis of several lung conditions in children. After stratification for sex, these associations were significant for asthma, bronchitis and number of lung infections for girls but not boys, whereas for lung infection, associations were significant for boys but not girls. These sex-specific associations may be related to known differences between males and females regarding development of the lungs and lung disorders. For instance, boys are more likely to develop asthma than girls, whereas adult females have a higher prevalence than adult males (37), suggesting the involvement of sex hormones. Furthermore, neonate boys have a higher chance of mortality due to respiratory distress syndrome than neonate girls (38). Next to males and females having different lung development, differences may also occur in the way male and female offspring respond to changes in maternal diet during pregnancy, as we have shown previously (39). Future studies will have to confirm whether indeed the relation between offspring lung disorders and maternal FA concentrations and specifically Mead acid concentrations differ between male and female offspring, and if so, what the underlying mechanisms are.

When interpreting results from the current study, it should be considered that the MEFAB cohort comprises a relatively healthy population. Although this consistency within the cohort enhances the comparability within participants, it also limits the applicability of the findings to less healthy populations, such as people with obesity. Furthermore, the MEFAB cohort has a relatively homogeneous genetic background, with 99% of the participants in the current analysis being Caucasian. Given the differences that exist in inflammatory markers between ethnicities (40, 41), the associations reported in this study may thus have been different if the ethnic background of the population would have been more diverse. Moreover, solely studying Caucasian participants in biomedical research contributes to health disparity. Future studies should therefore include other ethnicities as well, as did Rucci et al., who’s Generation R cohort consisted of 66% Europeans and 34% non-Europeans (17). Finally, health outcome parameters were assessed at the age of 7 years, and there was no control over the child’s lifestyle/environment between birth and the age of 7 years. In other words, although we observed a correlation between early life exposures and diseases later in life, it cannot be excluded that these are additionally related to exposures/lifestyle after birth.

In conclusion, our findings support the previously reported relation between inflammatory lung conditions in childhood and maternal fatty acid status during pregnancy. Furthermore, this work demonstrated the application of CCA within this field of research, which may lead to future studies using a similar approach. Lastly, future studies should investigate whether the importance of Mead acid in the relation between maternal FA status and child inflammation is due to its role as biomarker for essential fatty acid deficiency, due to its own biological function as pro-inflammatory mediator, or due to a combination of both.

## Data availability statement

The data analyzed in this study is subject to the following licenses/restrictions: data can be made available by sending email to corresponding author. Requests to access these datasets should be directed to marij.gielen@maastrichtuniversity.nl.

## Ethics statement

The studies involving humans were approved by Medical Ethics Committee, University Hospital, Maastricht/University of Maastricht, Netherlands. The studies were conducted in accordance with the local legislation and institutional requirements. Written informed consent for participation in this study was provided by the participants’ legal guardians/next of kin.

## Author contributions

SR: Formal analysis, Investigation, Methodology, Project administration, Visualization, Writing – original draft, Writing – review & editing. AS: Formal analysis, Methodology, Supervision, Writing – review & editing. MG: Conceptualization, Data curation, Project administration, Resources, Writing – review & editing. ReG: Investigation, Writing – review & editing. MZ: Funding acquisition, Investigation, Methodology, Resources, Supervision, Writing – review & editing. AO: Conceptualization, Funding acquisition, Investigation, Methodology, Supervision, Writing – review & editing. FS: Conceptualization, Investigation, Resources, Supervision, Writing – review & editing. RoG: Conceptualization, Investigation, Writing – original draft, Writing – review & editing.
