# Supplementary material for: Maternal fatty acid status during pregnancy versus offspring inflammatory markers: a canonical correlation analysis of the MEFAB cohort
Source: Front Nutr. 2023 Oct 19;10:1264278. doi: 10.3389/fnut.2023.1264278 (PMC10620499; doi:10.3389/fnut.2023.1264278)
Supplement: Supplementary file 1 [file Data_Sheet_1.pdf]

SUPPLEMENT

Maternal fatty acid status during pregnancy versus offspring inflammatory markers: a canonical correlation analysis of the MEFAB cohort

Sven H. Rouschop[1], Agnieszka Smolinska[1], Marij Gielen[2], Renate H. M. de Groot[3], Maurice P. Zeegers[2], Antoon Opperhuizen[1,4], Frederik J. van Schooten[1], Roger W. Godschalk[1*]

Table S1. List of the 39 fatty acids that were measured in maternal plasma at 16, 22 and 32 weeks of pregnancy, and at day of birth (in mg/L). DMA – dimethyl acetal, tr – truncated.

		16 weeks			22 weeks			32 weeks			Partus		
	Fatty acid	Mean		sd	Mean		sd	Mean		sd	Mean		sd
Saturated	14 :0	4.10	±	1.66	5.58	±	1.95	5.99	±	1.92	5.34	±	1.76
	15 :0	2.54	±	0.99	3.22	±	1.04	3.27	±	0.99	3.12	±	1.14
	16 :0	357.05	±	71.66	462.54	±	75.29	527.97	±	74.70	550.72	±	95.22
	17 :0	4.69	±	1.69	5.26	±	1.85	5.30	±	1.83	4.94	±	1.81
	18 :0	155.21	±	26.78	169.22	±	24.69	177.49	±	25.11	171.39	±	28.69
	20 :0	6.80	±	1.58	8.31	±	1.64	9.30	±	1.94	9.37	±	2.03
	22 :0	20.75	±	4.17	25.46	±	5.20	27.72	±	5.71	26.72	±	5.81
	23 :0	7.66	±	1.73	9.72	±	1.95	10.28	±	2.11	9.99	±	2.28
	24 :0	13.86	±	3.09	16.70	±	3.68	17.28	±	3.80	16.72	±	4.02
n-7	16 :1n-7	3.38	±	1.96	5.03	±	2.87	6.52	±	3.39	8.09	±	3.99
	18 :1n-7	20.60	±	7.43	24.76	±	8.35	25.86	±	8.75	25.31	±	8.70
	20 :1n-7	0.39	±	0.23	0.49	±	0.29	0.54	±	0.30	0.54	±	0.29
n-9	18 :1n-9	101.91	±	23.22	131.00	±	27.40	150.25	±	26.98	158.71	±	34.74
	18 :2n-9	0.41	±	0.24	0.41	±	0.28	0.40	±	0.24	0.34	±	0.24
	20 :1n-9	2.14	±	0.62	2.72	±	0.65	2.81	±	0.63	2.53	±	0.64
	20 :3n-9	2.56	±	1.46	3.31	±	1.72	3.73	±	1.89	4.31	±	2.48
	22 :1n-9	0.36	±	0.26	0.38	±	0.27	0.45	±	0.28	0.53	±	0.30
	22 :3n-9	0.06	±	0.11	0.08	±	0.12	0.07	±	0.12	0.09	±	0.14
	24 :1n-9	21.18	±	5.42	25.69	±	6.11	27.98	±	6.66	28.57	±	7.18
n-6	18 :2n-6	282.19	±	56.38	349.74	±	60.10	389.22	±	57.77	370.52	±	65.70
	18 :3n-6	0.47	±	0.36	0.55	±	0.42	0.59	±	0.43	0.61	±	0.43
	20 :2n-6	6.26	±	1.70	8.82	±	1.85	8.91	±	1.75	8.06	±	2.10
	20 :3n-6	41.68	±	12.39	54.49	±	13.47	60.23	±	13.72	61.98	±	15.57
	20 :4n-6	128.80	±	29.28	139.89	±	29.45	145.91	±	28.97	150.61	±	37.80
	22 :2n-6	0.20	±	0.16	0.27	±	0.26	0.32	±	0.19	0.36	±	0.20
	22 :4n-6	5.23	±	1.41	6.37	±	1.68	6.66	±	1.66	6.72	±	1.91
	22 :5n-6	4.67	±	1.78	7.34	±	2.59	8.43	±	2.81	9.38	±	3.65
	24 :2n-6	2.25	±	0.73	2.73	±	0.93	2.94	±	1.05	3.05	±	1.13
n-3	18 :3n-3	2.92	±	2.09	3.98	±	2.05	4.34	±	1.88	3.76	±	1.88
	20 :3n-3	0.45	±	0.27	0.58	±	0.30	0.52	±	0.27	0.41	±	0.30
	20 :4n-3	1.91	±	1.02	2.47	±	1.33	2.54	±	1.12	2.46	±	1.09
	20 :5n-3	6.96	±	4.82	6.88	±	7.06	6.29	±	3.60	5.92	±	3.07
	22 :3n-3	0.02	±	0.18	0.01	±	0.10	0.01	±	0.09	0.01	±	0.08
	22 :5n-3	10.02	±	2.89	10.30	±	3.06	10.30	±	2.70	10.03	±	2.82
	22 :6n-3	54.18	±	15.56	67.08	±	17.00	71.52	±	16.18	69.56	±	19.08
Derivatives	16 :0-DMA	8.86	±	2.37	9.06	±	2.35	8.73	±	1.85	8.43	±	2.08
	18 :0-DMA	6.51	±	2.33	6.44	±	2.09	6.38	±	1.89	5.44	±	1.87
	18 :1-DMA	2.38	±	0.81	2.45	±	0.79	2.27	±	0.69	2.01	±	0.63
	16 :1n-7tr	0.55	±	0.52	0.79	±	0.71	0.95	±	0.75	0.79	±	0.55

Note: dimethylacetals (DMA) may be products of the transesterification under acidic conditions. It has been reported that these fatty acid derivatives are formed from plasmalogens. Therefore, the amount of DMA fatty acids may reflect the concentration of plasmalogens.

Table S2. Canonical variates of canonical correlation analysis of maternal plasma fatty acid (FA) concentrations at 16, 22 and 32 weeks of pregnancy, and at day of birth (partus) versus child plasma inflammatory markers at seven years of age (n = 173 mother-child pairs). r, canonical correlation coefficient; p, p-value.

	**Week 16**		**Week 22**		**Week 32**		**Partus**	
**Canonical variate**	**r**	**p**	**r**	**p**	**r**	**p**	**r**	**p**
1	0.60	<0.001	0.62	0.000	0.64	0.001	0.61	0.028
2	0.45	0.108	0.48	0.194	0.44	0.431	0.46	0.594
3	0.43	0.305	0.41	0.675	0.41	0.759	0.43	0.823
4	0.38	0.588	0.35	0.918	0.35	0.943	0.41	0.951
5	0.34	0.785	0.32	0.983	0.33	0.983	0.35	0.995
6	0.33	0.893	0.22	0.998	0.25	0.998	0.28	0.999
7	0.25	0.980	0.19	0.997	0.23	0.999	0.26	0.999
8	0.22	0.982	0.16	0.994	0.16	1.000	0.21	0.999
9	0.18	0.984	0.14	0.984	0.10	1.000	0.18	0.997
10	0.09	0.987	0.08	0.967	0.08	0.989	0.14	0.982

Table S3. Associations between maternal plasma mead acid concentration at day of birth (mg/L) and self-reported doctor diagnosis of lung conditions in the child during the first seven years of age, adjusted for various population characteristics (n = 173 mother-child pairs). Statistical analysis by binomial logistic regression (for asthma, bronchitis and lung infection) or ordinal logistic regression (for number of lung conditions). OR, odds ratio; 95% CI, 95% confidence interval.

	OR	95% CI	p-value
**Asthma**			
Unadjusted	1.13	0.91 – 1.38	0.228
Maternal age	1.13	0.91 – 1.38	0.241
Maternal BMI	1.16	0.93 – 1.42	0.166
Gestational age	1.11	0.88 – 1.37	0.328
Smoking pregnancy	1.13	0.90 – 1.38	0.242
Alcohol pregnancy	1.13	0.90 – 1.37	0.260
Birthweight	1.13	0.90 – 1.38	0.241
Breastfeeding	1.15	0.92 – 1.40	0.198
Pets	1.13	0.91 – 1.38	0.217
Daycare attendance	1.13	0.91 – 1.38	0.235
Child BMI	1.12	0.90 – 1.37	0.265
**Lung infection**			
Unadjusted	1.27	1.04 – 1.55	0.014
Maternal age	1.31	1.07 – 1.62	0.009
Maternal BMI	1.27	1.04 – 1.55	0.016
Gestational age	1.26	1.04 – 1.54	0.018
Smoking pregnancy	1.27	1.04 – 1.55	0.014
Alcohol pregnancy	1.29	1.06 – 1.58	0.013
Birthweight	1.27	1.04 – 1.55	0.015
Breastfeeding	1.26	1.03 – 1.54	0.018
Pets	1.27	1.04 – 1.55	0.014
Daycare attendance	1.27	1.04 – 1.54	0.016
Child BMI	1.27	1.04 – 1.54	0.017
**Bronchitis**			
Unadjusted	1.17	1.01 – 1.34	0.030
Maternal age	1.17	1.02 – 1.35	0.024
Maternal BMI	1.18	1.03 – 1.37	0.020
Gestational age	1.15	1.00 – 1.32	0.043
Smoking pregnancy	1.19	1.04 – 1.38	0.013
Alcohol pregnancy	1.20	1.04 – 1.38	0.013
Birthweight	1.16	1.01 – 1.34	0.034
Breastfeeding	1.17	1.02 – 1.35	0.024
Pets	1.17	1.01 – 1.34	0.030

Daycare attendance	1.16	1.01 – 1.34	0.034
Child BMI	1.15	1.00 – 1.33	0.043
**Number of lung disorders**			
Unadjusted	1.18	1.03 – 1.34	0.007
Maternal age	1.19	1.04 – 1.36	0.005
Maternal BMI	1.20	1.05 – 1.37	0.004
Gestational age	1.17	1.02 – 1.33	0.010
Smoking pregnancy	1.20	1.05 – 1.38	0.003
Alcohol pregnancy	1.21	1.05 – 1.38	0.003
Birthweight	1.18	1.03 – 1.34	0.006
Breastfeeding	1.19	1.04 – 1.35	0.006
Pets	1.18	1.03 – 1.35	0.007
Daycare attendance	1.18	1.03 – 1.34	0.008
Child BMI	1.17	1.03 – 1.34	0.009
